# Supplementary material for: Core Outcome Set for Clinical Trials of COVID-19 Based on Traditional Chinese and Western Medicine
Source: Front Pharmacol. 2020 May 25;11:781. doi: 10.3389/fphar.2020.00781 (PMC7265660; doi:10.3389/fphar.2020.00781)
Supplement: Supplementary file 1 [file Table_1.docx]

Supplement 1. The list of outcomes that achieved consensus in different stakeholders in the round 1 of Delphi survey

| **Outcomes** | **TCM experts** | **Western medicine experts** | **Nurses** | **Public** |
| --- | --- | --- | --- | --- |
| Mortality | √ | √ | × | √ |
| leukocyte | ？ | × | √ | √ |
| Lymphocyte | ？ | √ | √ | √ |
| Neutrophil | × | ？ | √ | √ |
| Biochemical outcomes | × | ？ | √ | √ |
| Prothrombin time | × | × | ？ | ？ |
| Activated partial thromboplastin time | × | ？ | ？ | √ |
| Fibrinogen | × | × | ？ | ？ |
| D-dimer | × | × | ？ | ？ |
| Platelet count | × | ？ | ？ | ？ |
| Erythrocyte sedimentation (ES) rate | × | × | √ | ？ |
| Rate of ES recovery | × | × | √ | ？ |
| Time of ES recovery | × | × | √ | ？ |
| Chest imaging | √ | √ | √ | √ |
| Time to chest imaging recovery | √ | √ | √ | √ |
| Pneumonia severity index | √ | √ | √ | √ |
| Duration of mechanical ventilation | √ | ？ | ？ | √ |
| Arterial blood gas analysis | √ | √ | √ | √ |
| Blood oxygen saturation | √ | √ | √ | √ |
| PaO2/FiO2 | √ | √ | √ | √ |
| Application of pulmonary surfactant | ？ | √ | √ | √ |
| Duration of extracorporeal membrane oxygenation | ？ | ？ | √ | √ |
| Pulmonary function | √ | ？ | √ | √ |
| Oxygen intake methods | ？ | √ | √ | ？ |
| Frequency of requirement for supplemental oxygen | ？ | √ | √ | √ |
| Rate of no supplemental oxygen requirement | ？ | ？ | ？ | ？ |
| Duration of supplemental oxygenation | ？ | ？ | ？ | √ |
| Frequency of requirement for mechanical ventilation | ？ | × | ？ | √ |
| Rate of mechanical ventilation | ？ | ？ | ？ | √ |
| Time of using assisted breathing | ？ | ？ | √ | √ |
| Improvement of respiratory rate | ？ | √ | ？ | √ |
| Time to normalization of respiratory rate | ？ | √ | ？ | √ |
| Time to dyspnea reported as mild | √ | ？ | × | √ |
| The incidence of dyspnea with low oxygen saturation level and high respiratory rate | ？ | ？ | √ | √ |
| The incidence of hypoxia | ？ | ？ | √ | √ |
| Blood oxygen saturation improvement rate | √ | √ | √ | √ |
| Improvement of lung HRCT score | ？ | √ | √ | √ |
| CURB-65 | ？ | ？ | √ | √ |
| Murray lung injury score | ？ | ？ | √ | √ |
| St Georges respiratory questionnaire | × | × | √ | ？ |
| The duration of intubation | ？ | √ | √ | √ |
| The number of times incubation | ？ | ？ | √ | √ |
| Time to cough reported as mild | ？ | ？ | √ | √ |
| ECG | × | × | √ | ？ |
| Myocardial enzymes | ？ | ？ | √ | ？ |
| Myoglobin | × | × | √ | √ |
| Heart function | × | ？ | √ | √ |
| Rate of CK recovery | × | × | √ | √ |
| Time of CK recovery | × | × | √ | √ |
| Rate of Mb recovery | × | × | √ | √ |
| Time of Mb recovery | × | × | √ | √ |
| Troponin | × | × | √ | √ |
| Liver function | × | × | √ | √ |
| Rate of ALT recovery | × | × | √ | ？ |
| Time to ALT recovery | × | × | √ | ？ |
| Clearance time of cough | √ | × | √ | √ |
| Clearance time of fatigue | √ | × | √ | √ |
| Clearance time of fever | √ | √ | √ | √ |
| Clearance time of dyspnea | √ | √ | √ | √ |
| Clearance time of gastrointestinal symptoms | ？ | × | √ | ？ |
| Clearance time of myalgia | ？ | × | √ | ？ |
| Proportion of patients without cough | √ | × | √ | √ |
| Proportion of patients without fatigue | √ | × | √ | √ |
| Proportion of patients without fever | √ | √ | √ | √ |
| Proportion of patients without dyspnea | √ | ？ | √ | √ |
| Proportion of patients without sputum | √ | ？ | √ | √ |
| Proportion of patients without wheezing | √ | √ | √ | √ |
| Vital signs | ？ | ？ | √ | √ |
| TCM syndrome | ？ | × | √ | √ |
| Clinical symptom score | ？ | ？ | √ | √ |
| Duration of fever | ？ | ？ | √ | √ |
| Immunoglobulin | ？ | × | √ | √ |
| CD4+ T cell count | ？ | ？ | √ | √ |
| Time to CD4+ T cell recovery | ？ | ？ | √ | √ |
| CD8+ T cell count | ？ | ？ | √ | √ |
| Time to CD8+ T cell recovery | ？ | × | √ | √ |
| Human leukocyte antigen-DR | × | × | √ | √ |
| Rate of subjects receiving systematic corticosteroids | × | × | √ | √ |
| Lymphocyte subsets and complement | ？ | × | √ | √ |
| Recovery time of Lymphocyte subsets | × | ？ | √ | √ |
| C-reaction protein | ？ | √ | √ | √ |
| Time of CRP recovery | ？ | ？ | √ | √ |
| High-sensitive CRP | ？ | √ | √ | √ |
| Procalcitonin | ？ | × | √ | ？ |
| IL-2 | × | × | √ | √ |
| IL-4 | × | × | √ | ？ |
| IL-6 | ？ | ？ | √ | √ |
| IL-8 | × | × | √ | ？ |
| IL-10 | × | × | √ | ？ |
| γ-interferon | × | ？ | √ | √ |
| TNF-α | × | ？ | √ | √ |
| Time taken by SARS-CoV-2 RNA to become negative | ？ | √ | √ | √ |
| Proportion of patients with negative SARS-CoV-2 | ？ | √ | √ | √ |
| Declining speed of SARS-CoV-2 | ？ | √ | √ | √ |
| Incidence of antibiotic treatment | × | ？ | √ | √ |
| Duration of antibiotic treatment | × | × | √ | ？ |
| Level of viral antibody in blood sample | ？ | ？ | √ | √ |
| Other infection | × | ？ | √ | √ |
| Urine routine | × | × | √ | ？ |
| Kidney function | × | ？ | √ | √ |
| Incidence rate of kidney damage | × | ？ | √ | √ |
| APACHE II | × | ？ | √ | ？ |
| SOFA score | ？ | ？ | √ | √ |
| 6-minute walk test | × | × | ？ | √ |
| 7-point scale | × | × | √ | ？ |
| Modified Barthel Index | × | × | √ | ？ |
| NEWS2 | × | × | √ | ？ |
| Time to NEWS2 of ≤ 2 maintained for 24 h | × | × | √ | ？ |
| Incidence of multiple organ dysfunction | ？ | ？ | √ | √ |
| Rate of preventing mild to moderate type patients from progressing to severe type | √ | √ | √ | ？ |
| Complications | √ | √ | √ | √ |
| Incidence of shock | ？ | √ | √ | √ |
| DIC | ？ | √ | √ | √ |
| Major organ function | ？ | √ | √ | √ |
| Organ function support measures | ？ | √ | √ | √ |
| Organ support intensity | ？ | √ | √ | √ |
| Clinical outcome | √ | √ | √ | √ |
| Rate of disease remission | √ | √ | √ | ？ |
| Rate of severe type of disease | √ | √ | √ | ？ |
| The rate of critical stage | √ | √ | √ | √ |
| Time to severe stage | √ | √ | √ | √ |
| Time to the critical stage | √ | √ | √ | √ |
| Time to treatment failure | ？ | √ | √ | √ |
| Rate of preventing mild to moderate type patients from progressing to severe type | √ | √ | √ | √ |
| Number of participants with improvement from severe type to ordinary type | √ | √ | √ | ？ |
| Time to disease recovery | √ | √ | √ | √ |
| Recovery rate | √ | √ | √ | √ |
| Time to Clinical Improvement | √ | √ | √ | √ |
| Time to release from isolation | ？ | √ | √ | √ |
| Incidence of ICU admission | ？ | √ | √ | √ |
| Length of stay in ICU | ？ | √ | √ | √ |
| ICU free days | ？ | ？ | √ | √ |
| Duration of hospitalization | ？ | √ | √ | √ |
| The proportion of inpatients | × | ？ | √ | ？ |
| Hospitalization costs | × | × | √ | ？ |
| Demand for first aid measuments | ？ | ？ | √ | √ |
| Hemodiafiltration | × | × | √ | √ |
| Adverse events | ？ | ？ | √ | √ |
| CT of hip | × | × | ？ | ？ |
| MRI of hip | × | × | ？ | ？ |
| EQ-5D | × | ？ | ？ | ？ |
| SF-36 | × | ？ | ？ | ？ |
| Psychological outcomes | ？ | √ | √ | ？ |
| Liquid balance | ？ | ？ | √ | ？ |
| The rate of discontinuations due to adverse events | ？ | √ | √ | √ |

√: consensus in

×: consensus out

?: no consensus
