# Supplementary material for: Core Outcome Set for Clinical Trials of COVID-19 Based on Traditional Chinese and Western Medicine
Source: Front Pharmacol. 2020 May 25;11:781. doi: 10.3389/fphar.2020.00781 (PMC7265660; doi:10.3389/fphar.2020.00781)
Supplement: Supplementary file 2 [file Table_2.docx]

Supplement 2. The list of outcomes that achieved consensus in different stakeholders in the round 2 of Delphi survey

| **Outcomes** | **TCM experts** | **Western medicine experts** | **Nurses** | **Public** |
| --- | --- | --- | --- | --- |
| Mortality | √ | √ | √ | √ |
| leukocyte | √ | √ | √ | ? |
| Lymphocyte | √ | √ | √ | √ |
| Neutrophil | √ | ? | √ | √ |
| Biochemical outcomes | √ | ? | √ | √ |
| Prothrombin time | × | ? | √ | × |
| Activated partial thromboplastin time | × | ? | √ | √ |
| Fibrinogen | × | × | √ | ? |
| D-dimer | × | ? | √ | √ |
| Platelet count | × | ? | √ | √ |
| Erythrocyte sedimentation (ES) rate | × | × | √ | × |
| Rate of ES recovery | × | × | √ | × |
| Time of ES recovery | × | × | ? | × |
| Chest imaging | √ | √ | √ | √ |
| Time to chest imaging recovery | √ | √ | √ | √ |
| Pneumonia severity index | √ | √ | √ | √ |
| Duration of mechanical ventilation | √ | √ | √ | √ |
| Arterial blood gas analysis | √ | √ | √ | √ |
| Blood oxygen saturation | √ | √ | √ | √ |
| PaO2/FiO2 | √ | √ | √ | √ |
| Application of pulmonary surfactant | √ | ? | √ | √ |
| Duration of extracorporeal membrane oxygenation | √ | √ | √ | √ |
| Pulmonary function | √ | √ | √ | √ |
| Oxygen intake methods | √ | √ | √ | √ |
| Frequency of requirement for supplemental oxygen | √ | √ | √ | √ |
| Rate of no supplemental oxygen requirement | √ | √ | √ | √ |
| Duration of supplemental oxygenation | √ | √ | √ | √ |
| Frequency of requirement for mechanical ventilation | √ | √ | √ | √ |
| Rate of mechanical ventilation | √ | √ | √ | √ |
| Time of using assisted breathing | √ | √ | √ | √ |
| Improvement of respiratory rate | √ | √ | √ | √ |
| Time to normalization of respiratory rate | √ | √ | √ | √ |
| Time to dyspnea reported as mild | √ | ? | √ | √ |
| The incidence of dyspnea with low oxygen saturation level and high respiratory rate | √ | √ | √ | √ |
| The incidence of hypoxia | √ | √ | √ | √ |
| Blood oxygen saturation improvement rate | √ | √ | √ | √ |
| Improvement of lung HRCT score | √ | √ | √ | √ |
| CURB-65 | √ | ? | √ | ? |
| Murray lung injury score | √ | ? | √ | √ |
| St Georges respiratory questionnaire | × | × | √ | × |
| The duration of intubation | √ | ? | √ | √ |
| The number of times incubation | √ | √ | √ | ? |
| Time to cough reported as mild | √ | × | √ | √ |
| ECG | × | × | √ | × |
| Myocardial enzymes | √ | × | √ | √ |
| Myoglobin | × | ? | √ | ? |
| Heart function | ? | ? | √ | √ |
| Rate of CK recovery | × | × | √ | × |
| Time of CK recovery | × | × | √ | × |
| Rate of Mb recovery | × | × | √ | × |
| Time of Mb recovery | × | × | √ | × |
| Troponin | × | ? | ? | × |
| Liver function | × | × | √ | × |
| Rate of ALT recovery | × | × | √ | × |
| Time to ALT recovery | × | × | ? | × |
| Clearance time of cough | √ | × | √ | √ |
| Clearance time of fatigue | √ | × | √ | √ |
| Clearance time of fever | √ | √ | √ | √ |
| Clearance time of dyspnea | √ | √ | √ | √ |
| Clearance time of gastrointestinal symptoms | √ | × | √ | √ |
| Clearance time of myalgia | ？ | × | √ | √ |
| Proportion of patients without cough | √ | ？ | √ | √ |
| Proportion of patients without fatigue | √ | × | √ | √ |
| Proportion of patients without fever | √ | ？ | √ | √ |
| Proportion of patients without dyspnea | √ | × | √ | √ |
| Proportion of patients without sputum | √ | ？ | √ | √ |
| Proportion of patients without wheezing | √ | ？ | √ | √ |
| Vital signs | √ | √ | √ | √ |
| TCM syndrome | √ | ？ | √ | √ |
| Clinical symptom score | √ | ？ | √ | √ |
| Duration of fever | √ | √ | √ | √ |
| Immunoglobulin | ？ | × | √ | √ |
| CD4+ T cell count | ？ | ？ | ？ | √ |
| Time to CD4+ T cell recovery | ？ | ？ | √ | √ |
| CD8+ T cell count | ？ | ？ | √ | √ |
| Time to CD8+ T cell recovery | ？ | ？ | √ | √ |
| Human leukocyte antigen-DR | × | × | √ | ？ |
| Rate of subjects receiving systematic corticosteroids | ？ | × | √ | × |
| Lymphocyte subsets and complement | ？ | ？ | √ | ？ |
| Recovery time of Lymphocyte subsets | ？ | × | √ | ？ |
| C-reaction protein | √ | √ | √ | √ |
| Time of CRP recovery | √ | √ | √ | √ |
| High-sensitive CRP | √ | ？ | √ | ？ |
| Procalcitonin | ？ | ？ | √ | ？ |
| IL-2 | × | × | √ | × |
| IL-4 | × | × | √ | × |
| IL-6 | ？ | ？ | √ | √ |
| IL-8 | × | × | ？ | × |
| IL-10 | × | × | ？ | ？ |
| γ-interferon | × | × | ？ | ？ |
| TNF-α | × | × | ？ | × |
| Time taken by SARS-CoV-2 RNA to become negative | √ | √ | √ | √ |
| Proportion of patients with negative SARS-CoV-2 | √ | √ | √ | √ |
| Declining speed of SARS-CoV-2 | √ | √ | √ | √ |
| Incidence of antibiotic treatment | ？ | √ | √ | √ |
| Duration of antibiotic treatment | × | × | √ | ？ |
| Level of viral antibody in blood sample | √ | √ | √ | √ |
| Other infection | ？ | ？ | √ | √ |
| Urine routine | × | × | √ | × |
| Kidney function | ？ | × | √ | × |
| Incidence rate of kidney damage | ？ | × | √ | × |
| APACHE II | ？ | ？ | √ | √ |
| SOFA score | √ | ？ | √ | √ |
| 6-minute walk test | ？ | × | √ | ？ |
| 7-point scale | × | × | √ | × |
| Modified Barthel Index | × | × | √ | × |
| NEWS2 | × | × | √ | × |
| Time to NEWS2 of ≤ 2 maintained for 24 h | × | × | √ | × |
| Incidence of multiple organ dysfunction | √ | × | √ | √ |
| Rate of preventing mild to moderate type patients from progressing to severe type | √ | ？ | √ | √ |
| Complications | √ | ？ | √ | √ |
| Incidence of shock | √ | ？ | √ | √ |
| DIC | √ | ？ | √ | √ |
| Major organ function | √ | ？ | √ | √ |
| Organ function support measures | √ | ？ | √ | √ |
| Organ support intensity | √ | ？ | √ | √ |
| Clinical outcome | √ | √ | √ | √ |
| Rate of disease remission | √ | ？ | √ | √ |
| Rate of severe type of disease | √ | √ | √ | √ |
| The rate of critical stage | √ | √ | √ | √ |
| Time to severe stage | √ | √ | √ | √ |
| Time to the critical stage | √ | √ | √ | √ |
| Time to treatment failure | √ | ？ | √ | √ |
| Rate of preventing mild to moderate type patients from progressing to severe type | √ | √ | √ | √ |
| Number of participants with improvement from severe type to ordinary type | √ | √ | √ | √ |
| Time to disease recovery | √ | √ | √ | √ |
| Recovery rate | √ | √ | √ | √ |
| Time to Clinical Improvement | √ | √ | √ | √ |
| Time to release from isolation | √ | √ | √ | √ |
| Incidence of ICU admission | √ | √ | √ | √ |
| Length of stay in ICU | √ | √ | √ | √ |
| ICU free days | √ | √ | √ | √ |
| Duration of hospitalization | √ | √ | √ | √ |
| The proportion of inpatients | ？ | ？ | √ | √ |
| Hospitalization costs | × | ？ | √ | × |
| Demand for first aid measuments | √ | √ | √ | √ |
| Hemodiafiltration | ？ | ？ | √ | × |
| Adverse events | √ | √ | √ | √ |
| Hip imaging | × | × | ？ | × |
| EQ-5D | × | × | √ | × |
| SF-36 | ? | × | √ | × |
| Psychological outcomes | √ | ? | √ | √ |
| Liquid balance | √ | √ | √ | √ |
| The rate of discontinuations due to adverse events | √ | √ | √ | √ |
| Quality of sleep | ? | ? | √ | √ |
| Decline of temperature | ? | ? | √ | √ |
| Area of temperature declining and time | ? | ? | √ | ? |
| Tongue coating and pulse | √ | ? | √ | √ |
| Diet | √ | ? | √ | √ |
| Viral load | √ | √ | √ | √ |

√: consensus in

×: consensus out

?: no consensus
