# Supplementary material for: Core Outcome Set for Clinical Trials of COVID-19 Based on Traditional Chinese and Western Medicine
Source: Front Pharmacol. 2020 May 25;11:781. doi: 10.3389/fphar.2020.00781 (PMC7265660; doi:10.3389/fphar.2020.00781)
Supplement: Supplementary file 3 [file Table_3.docx]

Supplement 3 Outcomes/outcome domains in patients’ questionnaire

Mortality

Blood routine test

Urine routine test

Coagulation

Erythrocyte sedimentation (ES) rate

Chest imaging

Dyspnea

Mechanical ventilation usage

Extracorporeal membrane oxygenation usage

Pulmonary function

Heart function

Liver function

Kidney function

ECG

Myocardial enzymes

Respiratory symptoms

Fever

Fatigue

Gastrointestinal symptoms

Myalgia

Vital signs

Immunological outcomes

Inflammatory factor

SARS-CoV-2 nucleic acid tests

Viral antibody

Complications

Other infection

Incidence of antibiotic treatment

Organ function

Rate of severe/critical type of disease

Rate of disease remission

Recovery rate

Time to release from isolation

Incidence and length of ICU admission

Duration of hospitalization

Hospitalization costs

Demand for first aid measuments

Adverse events

Hip imaging

Quality of life

Mental

Liquid balance

The rate of discontinuations due to adverse events
