# Supplementary material for: Core Outcome Set for Clinical Trials of COVID-19 Based on Traditional Chinese and Western Medicine
Source: Front Pharmacol. 2020 May 25;11:781. doi: 10.3389/fphar.2020.00781 (PMC7265660; doi:10.3389/fphar.2020.00781)
Supplement: Supplementary file 4 [file Table_4.docx]

Supplement 4 The voting results of consensus meeting

| **Outcomes** | **Voting results** |
| --- | --- |
| Clinical outcome (Recovery, improvement, progression, death) | 9 (9/9, 100%) |
| SARS-CoV-2 nucleic acid tests | 9 (9/9, 100%) |
| Viral load | 7 (7/9, 77.78%) |
| C-reaction protein | 8 (8/9, 88.89%) |
| Temperature | 8 (8/9, 88.89%) |
| Breathing | 8 (8/9, 88.89%) |
| Adverse events | 6 (6/9, 66.67%) |
| The rate of discontinuations due to adverse events | 4 (4/9, 44.44%) |
| Lymphocyte | 8 (8/9, 88.89%) |
| Level of viral antibody in blood sample | 7 (7/9, 77.78%) |
| Liquid balance | 0 |
| Chest imaging | 9 (9/9, 100%) |
| Arterial blood gas analysis | 8 (8/9, 88.89%) |
| Blood oxygen saturation | 7 (7/9, 77.78%) |
| PaO2/FiO2 | 8 (8/9, 88.89%) |
| Mechanical ventilation | 7 (7/9, 77.78%) |
| Oxygen intake | 7 (7/9, 77.78%) |
| The incidence of hypoxia | 6 (6/9, 66.67%) |
| Pulmonary function | 3 (3/9, 33.33%) |
| Pneumonia severity index | 7 (7/9, 77.78%) |
| extracorporeal membrane oxygenation | 6 (6/9, 66.67%) |
| Demand for first aid measuments | 6 (6/9, 66.67%) |
| Time to release from isolation | 5 (5/9, 55.56%) |
| ICU admission/ hospitalization | 6 (6/9, 66.67%) |
| ICU free days | 6 (6/9, 66.67%) |
| Rate of preventing mild to moderate type patients from progressing to severe type | 7 (7/9, 77.78%) |
| CD4+ T cell count | 2 (2/9, 22.22%) |
| Time to CD4+ T cell recovery | 1 (1/9, 11.11%) |
| CD8+ T cell count | 2 (2/9, 22.22%) |
| Time to CD8+ T cell recovery | 1 (1/9, 11.11%) |
| Immunoglobulin | 3 (3/9, 33.33%) |
| Lymphocyte subsets count | 4 (4/9, 44.44%) |
| Time of lymphocyte subsets recovery | 2 (2/9, 22.22%) |
| Time of complement recovery | 2 (2/9, 22.22%) |
| CURB-65 | 4 (4/9, 44.44%) |
| Murray lung injury score | 4 (4/9, 44.44%) |
| The duration and number of times of incubation | 0 |
| Application of pulmonary surfactant | 1 (1/9, 11.11%) |
| Coagulation | 3 (3/9, 33.33%) |
| leukocyte | 2 (2/9, 22.22%) |
| Neutrophil | 3 (3/9, 33.33%) |
| High-sensitive CRP | 3 (3/9, 33.33%) |
| Biochemical outcomes | 3 (3/9, 33.33%) |
| Complications | 2 (2/9, 22.22%) |
| 6-minute walk test | 2 (2/9, 22.22%) |
| 窗体顶端  APACHEII 窗体底端 | 2 (2/9, 22.22%) |
| SOFA score | 2 (2/9, 22.22%) |
| Organ function support measures and intensity | 2 (2/9, 22.22%) |
| Incidence of multiple organ dysfunction | 2 (2/9, 22.22%) |
| DIC | 2 (2/9, 22.22%) |
| Incidence of shock | 2 (2/9, 22.22%) |
| Time to treatment failure | 2 (2/9, 22.22%) |
| Psychological outcomes | 4 (4/9, 44.44%) |
| Other infection | 2 (2/9, 22.22%) |
| Rate of subjects receiving systematic corticosteroids | 2 (2/9, 22.22%) |
| Incidence of antibiotic treatment | 3 (3/9, 33.33%) |
| The proportion of inpatients | 2 (2/9, 22.22%) |
| Hospitalization costs | 2 (2/9, 22.22%) |
| Hemodiafiltration | 2 (2/9, 22.22%) |
| Kidney function | 2 (2/9, 22.22%) |
| Incidence rate of kidney damage | 2 (2/9, 22.22%) |
| 窗体顶端  SF-36 窗体底端 | 2 (2/9, 22.22%) |
| IL-6 | 4 (4/9, 44.44%) |
| Procalcitonin | 4 (4/9, 44.44%) |
| Proportion of patients without fever | 8 (8/9, 88.89%) |
| Proportion of patients without fatigue | 5 (5/9, 55.56%) |
| Proportion of patients without wheezing | 4 (4/9, 44.44%) |
| Proportion of patients without dyspnea | 8 (8/9, 88.89%) |
| Proportion of patients without cough | 6 (6/9, 66.67%) |
| Proportion of patients without sputum | 4 (4/9, 44.44%) |
| Clearance time of fatigue | 6 (6/9, 66.67%) |
| Clearance time of myalgia | 4 (4/9, 44.44%) |
| Clearance time of cough | 6 (6/9, 66.67%) |
| Clearance time of gastrointestinal symptoms | 4 (4/9, 44.44%) |
| Time to dyspnea/cough reported as mild | 6 (6/9, 66.67%) |
| Clinical symptom score | 7 (7/9, 77.78%) |
| Sleeping | 3 (3/9, 33.33%) |
| Area of temperature declining and time | 4 (4/9, 44.44%) |
| Decline of temperature | 3 (3/9, 33.33%) |
| Diet | 1 (1/9, 11.11%) |
| Tongue coating and pulse | 4 (4/9, 44.44%) |
| TCM syndrome | 5 (5/9, 55.56%) |
| Heart function | 3 (3/9, 33.33%) |
| Myocardial enzymes | 2 (2/9, 22.22%) |
| Troponin | 2 (2/9, 22.22%) |
